# Assessing aesthetic impressions with pictorial measures: A novel approach in empirical aesthetics

**DOI:** 10.1177/20416695241309780

**Published:** 2025-02-14

**Authors:** Ivan Z. Stojilović

**Affiliations:** Department of Psychology, Faculty of Philosophy, University of Pristina in Kosovska Mitrovica

**Keywords:** pictorial technique, aesthetic impressions, empirical aesthetics, visual art, pictorial measures

## Abstract

This study introduces pictorial technique (PT) as an innovative method in empirical aesthetics to assess aesthetic impressions of visual artworks. Forty participants, drawn from general and artistic populations, evaluated nine paintings representing abstract, traditional figural, and modern figural styles using the PT and aesthetic rating scales. The PT enabled participants to mark impactful areas within artworks, transforming subjective impressions into spatial data visualized as heatmaps. Results showed that, on average, participants marked 17% of the painting's surface, with notable stylistic differences in attention distribution. Abstract paintings exhibited dispersed attention, focusing on geometric shapes and color contrasts, while traditional figural works concentrated on narrative elements. Modern figural paintings demonstrated a hybrid pattern, emphasizing both individual details and broader compositions. The study also tested the hypothesis that dimensional characteristics of marked areas correspond to aesthetic preferences. Findings revealed that the size of marked regions modestly predicted ratings on Interestingness and Comprehensibility scales, though the explained variance was limited. The study highlights the PT's potential for visualizing aesthetic engagement and suggests its integration with physiological methods like eye-tracking to explore the interaction between spontaneous attention and reflective aesthetic judgments. These findings underscore PT's adaptability and value as a tool for investigating aesthetic experiences across diverse art forms and cultural contexts.

## How to cite this article

Ivan Z.S. (2025). Assessing aesthetic impressions with pictorial measures: A novel approach in empirical aesthetics. *i-Perception,* 16(0), 1–22. https://doi.org/10.1177/20416695241309780

Within the realm of art, the appreciation of an artwork varies among observers. Some individuals value the mastery and skill of the artist and the artwork, while others seek to experience a strong emotional response or gain a new perspective on the world (Csikszentmihalyi & Robinson, 1990; Leder et al., 2012). In the study of aesthetic constructs, various techniques and measures are employed, which can generally be categorized into two groups: objective and subjective methods (Kozbelt & Kaufman, 2014). Objective methods measure observable phenomena, whereas subjective methods involve self-reported psychological processes (Radonjić, 1981). The research methodology in empirical aesthetics is multifaceted and intricate, owing to the vast array of possible aesthetic objects, their complexity, and the diversity of their meanings. As a result, an assortment of complex and diverse methods must be utilized (Kozbelt & Kaufman, 2014).

In the realm of objective measures, among others, the archival method is included (Kozbelt, 2006; Martindale, 1990; Murray, 2003; Simonton, 2007), the study of the objective characteristics of the artwork (Firstov et al., 2007; Graham & Redies, 2010), physiological measures (de Tommaso et al., 2008; Francuz et al., 2018; Gerger & Leder, 2015; Krupinski & Locher, 1988; Locher, 2020; Nodine & Krupinski, 2003; Quiroga & Pedreira, 2011; Tröndle & Tschacher, 2012; Winkielman & Cacioppo, 2001), or observation (Carbon, 2017; Smith & Smith, 2001; Tinio & Specker, 2021; Tröndle & Tschacher, 2012). Subjective methods are used in the majority of empirical aesthetics studies (Kozbelt & Kaufman, 2014). These methods include interviews (Csikszentmihalyi & Robinson, 1990), the consensual assessment technique (Amabile, 1983; Baer et al., 2004; Beghetto & Kaufman, 2014; Hennessey & Amabile, 1999), different rating scales (Gerger & Leder, 2015; Newman, 2016; Specht, 2010; Wanzer et al., 2020), or free verbal descriptions (Augustin et al., 2012; Jacobsen et al., 2004), among others. Using these methods, we can explore constructs such as aesthetic preferences (feelings about the work), aesthetic impressions (results of aesthetic experience), aesthetic emotions (emotional responses produced by an aesthetic artifact), aesthetic judgments (evaluating the artifact as a work of art), or aesthetic experience (a complex internal process that involves perception, cognition, emotion, and evaluation). While these approaches have advanced our understanding of aesthetics, they often address distinct dimensions independently.

To bridge this gap, we introduce the pictorial technique (PT) as a tool for examining aesthetic impressions^1^. By capturing subjective responses as spatial data, the PT integrates the strengths of both objective and subjective methods, offering insights into aesthetic salience and individual preferences. The primary objective of this study is to evaluate the PT's utility and explore its relationship with aesthetic preferences derived from verbal rating scales.

### Introducing the Pictorial Technique

The PT enables participants to interact directly with artworks by identifying and marking the areas they find most visually, emotionally, or cognitively impactful. These markings are aggregated into heatmaps that visualize patterns of aesthetic salience across individuals. This transformation of subjective experiences into measurable data allows researchers to analyze the size, distribution, and properties of marked regions. PT generates outputs that reveal the “hotspots” of visual interest within an artwork, allowing researchers to explore individual and group preferences. For instance, this approach can help examine differences in visual focus between novices and art experts, as prior studies have demonstrated that novices often attend to representational elements, whereas experts focus on compositional or abstract features (Hekkert & Van Wieringen, 1996; Millis, 2001; Silvia, 2013; Stojilović & Marković, 2014). To the best of our knowledge, this represents the first application of this technique within the realm of empirical aesthetic studies.

### Comparison with Similar Techniques

The PT shares similarities with several established techniques for studying visual attention and aesthetic engagement but also offers unique advantages and distinctions. Below is a comparison with five related techniques:

1. *Eye-tracking*. The PT and eye-tracking share the goal of identifying areas of visual interest, but they differ significantly in their methodological approach. Eye-tracking captures spontaneous gaze patterns in real time, providing temporal data on how participants explore an artwork. This technique highlights the dynamic process of visual exploration, often beginning with an understanding of the compositional structure and semantic essence of a painting, before shifting to specific pictorial attributes of perceptual or semantic significance (Locher, 2006; Quiroga & Pedreira, 2011). Familiarity with paintings also influences gaze behavior, with experts and novices displaying distinct patterns. For instance, experts focus more on the overall structure and formal elements, while novices tend to fixate on representational features like objects or human figures (Francuz et al., 2018; Nodine et al., 1993; Vogt & Magnussen, 2007). In contrast, the PT involves a deliberate selection process, asking participants to actively mark the most impactful regions of a painting. This process reflects a considered judgment of aesthetic significance, capturing the reflective engagement of participants rather than transient attention shifts. While eye-tracking provides insights into immediate cognitive and perceptual processes, the PT complements this by focusing on the intentional aspects of aesthetic experience. Together, these techniques can offer a comprehensive understanding of how viewers engage with artworks, from spontaneous visual exploration to thoughtful evaluation.
2. *Photo cropping*. The PT bears some resemblance to photo cropping tasks, such as those used by McManus and colleagues (2011). In both techniques, participants engage directly with visual stimuli to identify or enhance areas of interest. However, there are notable differences in their objectives and applications. Photo cropping emphasizes aesthetic optimization, as participants adjust the composition of photographs to improve their visual appeal. In contrast, the PT maintains a focus on perception and experience, asking participants to highlight the elements of an artwork they find most engaging. This shift from creation to perception makes the PT better suited for studying aesthetic responses to pre-existing artworks.
3. *Objective measures of artworks*. Objective measures, such as calculating the colorimetric barycenter or analyzing Fourier spectral power (Firstov et al., 2007; Graham & Redies, 2010), focus on identifying compositional features of an artwork as designed by the artist. For example, the barycenter technique determines the geometric or color “center” of a painting, while spectral analysis explores fractal patterns and visual complexity. The PT diverges from these techniques by prioritizing the *perceptual center*—the area that participants find most impactful during their viewing experience. This distinction underscores the PT's strength in capturing the viewer's perspective rather than the objective properties of the artwork itself.
4. *Mouse tracking.* Heatmaps generated from interaction data, such as mouse clicks or hover patterns, are similar to the outputs of the PT, as both techniques produce visual representations of engagement. However, interaction-based heatmaps typically derive from spontaneous digital behavior, such as hovering over web elements (Navalpakkam & Churchill, 2012), while the PT involves intentional and focused interaction with artworks. The deliberate nature of the PT allows for a deeper exploration of aesthetic preferences, as participants actively reflect on what they find impactful in a painting. Additionally, while heatmaps are often used in UX design to optimize user interfaces, the PT is uniquely adapted to the context of aesthetic research.
5. *Bubbles technique*. The bubbles technique, like the PT, aims to uncover visual elements critical for specific tasks. In the bubbles technique, participants view stimuli where only parts of the image are visible through randomly placed apertures (“bubbles”), and their task performance determines which visual information is most diagnostic (Gosselin & Schyns, 2001). This technique is particularly suited for tasks like facial recognition or expression categorization. In contrast, the PT emphasizes aesthetic salience and engagement, allowing participants to freely select impactful areas rather than relying on pre-determined constraints. While the bubbles technique is more controlled and task-specific, the PT offers greater freedom for participants to express subjective impressions, making it better suited for studies of aesthetic experience.

### The Relationship Between Pictorial Measures and Aesthetic Preferences

In addition to establishing the validity of the PT in empirical aesthetics, our study aimed to examine the relationship between pictorial measures and aesthetic preferences obtained through Likert-type scale ratings. Pictorial measures derived from PT can be categorized into two groups: *dimensional* measures (e.g., area size, width, and height) and *chromatic* values. Based on philosophical, theoretical, and empirical findings, we hypothesized a positive relationship between the size of marked regions and aesthetic preferences.

Historically, spatial magnitude has been closely linked to aesthetic judgment. Philosophers have long explored the interplay between size and artistic value, particularly in the context of the sublime. Kant, in his *Critique of Judgment* (1790/2005), differentiated between the mathematical sublime, evoked by objects so vast that they challenge comprehension, and the dynamical sublime, which involves overwhelming natural forces. The mathematical sublime, in particular, compels the mind to transcend sensory limitations, evoking awe and grandeur. Similarly, Burke (1767/1958) argued that greatness of dimension is a key aspect of the sublime, producing feelings of astonishment and reverence. Mendelssohn added an emotional dimension to this perspective, describing immensity as an aesthetic quality that evokes a “sweet shudder,” blending pleasure and awe (Seidel & Prinz, 2018, p. 3). Hegel (1835/1998) emphasized spatial magnitude in monumental art, asserting that size reflects the grandeur of spiritual and cultural ideals. These historical perspectives collectively underscore the enduring association between spatial magnitude and aesthetic judgment.

Modern theories offer further insights into this relationship. According to Seidel and Prinz (2018), *embodied cognition* plays a central role in how humans interpret physical properties, such as size, through bodily experiences. Lakoff and Johnson's (1980) *conceptual metaphor theory* suggests that larger dimensions are metaphorically linked to notions of importance, value, and grandeur—concepts derived from everyday experiences where size often correlates with power or dominance, such as towering structures or expansive landscapes. Theories of *visual salience and attention* further contribute to this understanding, as larger objects are often more visually distinctive, attracting attention and enhancing their perceived importance (Huang & Pashler, 2005; Peschel & Orquin, 2013). Additionally, *awe-inducing experiences* triggered by large-scale stimuli can lead to self-diminishment and an expanded sense of the external environment, amplifying the aesthetic impact of monumental structures or artworks (Seidel & Prinz, 2018; Shiota et al., 2007). On the other hand, the “ecological” hypothesis suggests that the preferred size of any object (not limited to art objects) is influenced by its actual physical size in reality (Linsen et al., 2011).

Empirical findings do not provide unequivocal answers regarding the relationship between size and aesthetic preferences (Chu et al., 2013; Linsen et al., 2011; Seidel & Prinz, 2018; Silvera et al., 2002). For example, Seidel and Prinz (2018) demonstrated that increasing the size of a painting enhances its perceived aesthetic value, while artworks attributed to master artists were perceived as larger and closer than identical works attributed to students or forgers. These findings highlight a reciprocal relationship between spatial magnitude and aesthetic evaluation, wherein perceptions of size influence, and are influenced by, judgments of artistic quality. Conversely, Linsen et al. (2011) confirmed the canonical size effect, where physically smaller objects are more aesthetically preferred if depicted as smaller relative to a reference frame, while physically larger objects are preferred when depicted as larger relative to the same reference frame. Chu et al. (2013) further revealed that the size effect depends on the content of the object. It is important to note that the latter two studies utilized images of real-world objects, rather than artistic stimuli, as their focus.

While previous research has investigated the relationship between the physical size of aesthetic objects and their evaluation, our study sought to examine the connection between the size of marked regions deemed impactful and the aesthetic appeal of the painting. We hypothesized that, similar to Seidel and Prinz (2018) findings, there would be a positive correlation between the size (surface area) of marked regions and the aesthetic appeal of the painting, as expressed through verbal techniques.

## Method

### Participants

The sample comprised of 40 participants from two distinct groups. The first group included 27 participants selected through convenience sampling from the general population. Their age spanned from 20 to 67 years (*M* = 41.7, *SD* = 12.4), with 18 females among them. The second group comprised 13 fourth-year students from the Painting, Sculpture, and Graphics department at the Faculty of Fine Arts (FFA) in Belgrade. Selected through the same convenience sampling method, this group exhibited ages ranging from 18 to 34 years (*M* = 21, *SD* = 4.1), encompassing eight female participants. The FFA group had a much higher level of familiarity with the artworks compared to the general population group, suggesting a superior understanding of art history (*F*(1, 38) = 25.87, *p* < .001, *ω*^2^ = .38). The mean familiarity score for the general population was 1.57 (*SD* = 0.51), whereas for FFA it was 2.55 (*SD* = 0.68).

Prior to testing, all participants provided written informed consent to participate. The study adhered to the ethical principles delineated in the 1964 Helsinki Declaration and its subsequent revisions, or equivalent ethical standards, in conjunction with the institutional protocols outlined by the Faculty of Philosophy. The participation of individuals in the study safeguarded their physical and psychological well-being, privacy, and overall personal rights and interests.

### Stimuli

At the outset, the researcher initially curated a collection of 21 paintings, encompassing seven paintings of abstract style, seven of traditional figurative style from the Baroque period (16th and 17th centuries), and seven of contemporary figurative style from the late 20th and early 21st centuries. Abstract style pertains to visual artworks that lack mimetic or representational qualities.

To evaluate the alignment of each painting with its designated style, an online questionnaire was administered to three experts, including an art historian and two visual artists. A scale spanning from 1 = *Not belonging at all* to 5 = *Completely belonging* was employed for assessment. Only paintings unanimously endorsed by all three experts as fitting their respective styles were retained for the final selection. The ultimate collection of artistic pieces comprised of nine paintings: three of Abstract style, three of Figurative traditional style, and three of Figurative contemporary style. The definitive compilation of the selected paintings is detailed in Table 1 in the Supplemental materials.

The H/W percentage was calculated for all artworks to determine their orientation. A number greater than 1 suggests a horizontal orientation, a value of 1 corresponds to a square form, and values less than 1 indicate a vertical orientation. The range of values observed for the selected artworks varied from 0.68 to 1.61, with an average value of 1.14 (*SD* = 0.38). The aspect ratio did not vary significantly among the three painting techniques (*F*(2, 6) = 0.28, *p* = .77, *ω*^2^ = .00; see Table 1).

**Table 1.** Means and SDs (in parentheses) for proportion and lab values for used paintings.

	All paintings	Abstract	Figural contemporary	Figural traditional
Proportion (W/H)	1.14 (0.38)	0.99 (0.44)	1.22 (0.38)	1.21 (0.42)
*L*	54.06 (12.60)	60.78 (12.08)	61.65 (4.99)	39.76 (1.87)
*a*	11.15 (11.65)	19.80 (17.92)	7.27 (5.75)	6.38 (4.44)
*b*	19.35 (10.80)	30.03 (13.05)	15.34 (5.47)	12.69 (2.19)

The study also included the calculation of the average chromatic value for each painting using the CIE Lab color system. The CIE Lab color model defines colors based on their lightness and two additional components: “*a*” and “*b*.” The “*a*” component spans from green to red, while the “*b*” component ranges from blue to yellow. This color model is designed to align with human color perception and can effectively describe all colors visible to a person with normal vision. In this model, the “*L*” value represents lightness and varies from 0 (representing black) to 100 (representing diffuse white). Meanwhile, the “*a*” value ranges from −128 (indicating green) to +127 (indicating red), and the “*b*” value ranges from −128 (representing blue) to +127 (representing yellow). For all the paintings analyzed in the study, the average mean “*L*” (lightness) value was found to be 54. Notably, there were significant variations in “*L*” values observed between paintings of different styles (*F*(2, 6) = 7.94, *p* = .021, *ω*^2^ = .61). Specifically, Abstract and Figural contemporary paintings were notably brighter than the Traditional paintings that were examined. In terms of the “*a*” and “*b*” values, the mean “*a*” value was 11, and the mean “*b*” value was 19. There were no substantial differences observed in the “*a*” values between the various painting styles (*F*(2, 6) = 1.36, *p* = .327, *ω*^2^ = .07), and the same was true for the “*b*” values (*F*(2, 6) = 3.83, *p* = .085, *ω*^2^ = .17).

### Instruments

*Aesthetic Rating Scales.* In this study, three 7-point Likert rating scales (1 = *not at all*, 7 = *very much*) were employed to determine aesthetic impressions, namely interestingness, pleasantness, and comprehensibility. These three scales were selected to represent aesthetic outcomes in the conative, emotional, and cognitive domains. Each painting was also evaluated using another scale, namely the scale of Familiarity.

#### Verbal Descriptions of Aesthetic Impressions

Respondents were allowed to provide an unlimited number of verbal descriptions for each painting. The results of this analysis are not presented further in this study, as they are intended to serve as the basis for our future research.

### Equipment

All monitors were checked and standardized in terms of resolution, color, and brightness. The monitors used were of the same type (Asus PB287Qmonitor) with a response time of 1 ms, a screen refresh rate of 60 Hz, and were connected to the same computers (Fujitsu Lifebook A Series). Participants were positioned about 70 cm away from the monitor. The paintings on monitors are displayed with their original aspect ratio, ensuring they occupy the maximum available area on the screen.

### Procedure

Before the main research phase began, all participants had completed a basic socio-demographic questionnaire (to assess age and gender). The study was conducted in two blocks with smaller groups of two to five participants, each of whom had access to a personal computer to view the paintings and provide responses. In the first block, participants were exposed to nine paintings displayed in a randomized sequence on a monitor. Participants were asked to mark the most impressive part of the painting, with an emphasis on the freedom to use their own criteria for determining the most impressive part. There was no time limit for completing these activities.

In the second block, participants were asked to evaluate the same nine paintings in the same order, using three aesthetic scales and a Familiarity scale. All responses were provided using a computer. At the end of the second block, the researcher gave brief feedback on the research and thanked the participants. The complete procedure took approximately 50 min.

### Analytical Strategy

#### Pictorial Measures

**Figure 1.**
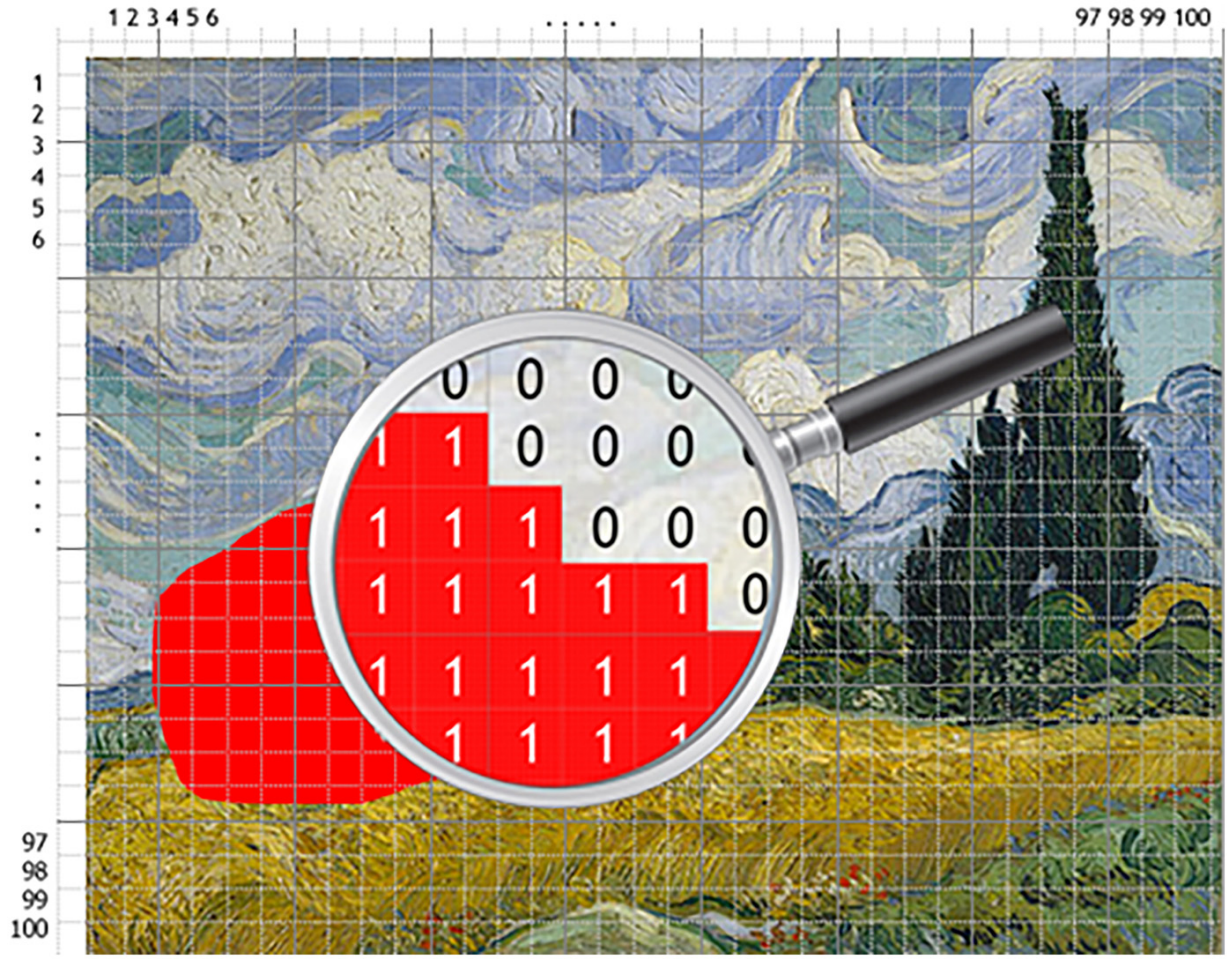
Example of painting mapping and area value determination.

In the first block, participants selected the part of each painting that impressed them the most by marking a corresponding area. Subsequently, each painting was divided into a 100 × 100 grid, and for each cell grid it was recorded whether it dominantly covered the marked part (value 1) or not (value 0) (refer to Figure 1 for a visual representation). Cells were squares for paintings with equal width and height; in other instances, cells were rectangles. The resulting matrix of areas, thus generated, was subsequently transformed into a linear array consisting of 10,000 data points, with each data point being assigned a value of either 0 or 1. To convert the visual content of the paintings into numerical data, we employed Photoshop scripts. This allowed us to calculate the area covered, as well as the width and height (relative to the painting's dimensions) for each of the marked areas. Additionally, we computed the average chromatic values for each marked area using the CIE Lab color system.

#### Interrater Reliability for Pictorial Measures

To determine the degree of agreement between respondents’ marked areas for nominal data, we utilized the generalized Fleiss’ kappa coefficient (*κ^*). This measure was specifically chosen for its applicability to dichotomous data (0 = *Not marked* and 1 = *Marked*). Fleiss’ kappa (*κ*) is designed to measure the extent of agreement beyond what would be expected by chance, as originally described by Fleiss (1971) and later elaborated by Shrout and Fleiss (1979) and Hallgren (2012). The range of Fleiss’ kappa is from −1 to +1. A negative kappa score signifies that the level of agreement among two or more respondents was lower than what would be predicted by chance. Alternatively, kappa values larger than 0 imply a rising level of agreement that is better than chance for two or more respondents. The highest value of +1 represents complete agreement, meaning that the raters agreed on everything. In practical terms, the interpretation of kappa values can be categorized as follows: values below 0.5 are generally considered indicative of poor agreement; values between 0.5 and 0.75 suggest moderate agreement; values from 0.75 to 0.9 indicate good agreement, and values above 0.9 are interpreted as evidence of excellent agreement.

#### Relationship Between Pictorial Measures and Aesthetic Preferences

Given that our variables are repeated measures, we opted for multivariate multilevel models, also known as hierarchical linear models or mixed models (Heck & Thomas, 2020; Hox, 2010; Preacher et al., 2010; Raudenbush & Bryk, 2002). These models enable us to evaluate the predictive validity of Pictorial measures against criteria comprising three aesthetic rating scales. All these measures were repeatedly assessed as participants viewed nine distinct paintings at level 1. We included three subject-level (level 2) variables as control variables: gender, age, and group membership (general or FFA group). These variables were introduced to account for potential confounding factors in our analysis. We employed a two-level hierarchical linear model, where pictorial and verbal aesthetic impressions (level 1) were nested within individuals (level 2). We utilized Mplus version 8.9, a statistical modeling software, to do multivariate multilevel modeling (Asparouhov & Muthén, 2010; Muthén, 2010). Maximum-likelihood robust estimation was used, which offers a robust and flexible approach to estimating parameters in multilevel models, particularly in situations where data may not meet the assumptions of traditional estimation methods.

## Results

### Visualization of PT

**Figure 2.**
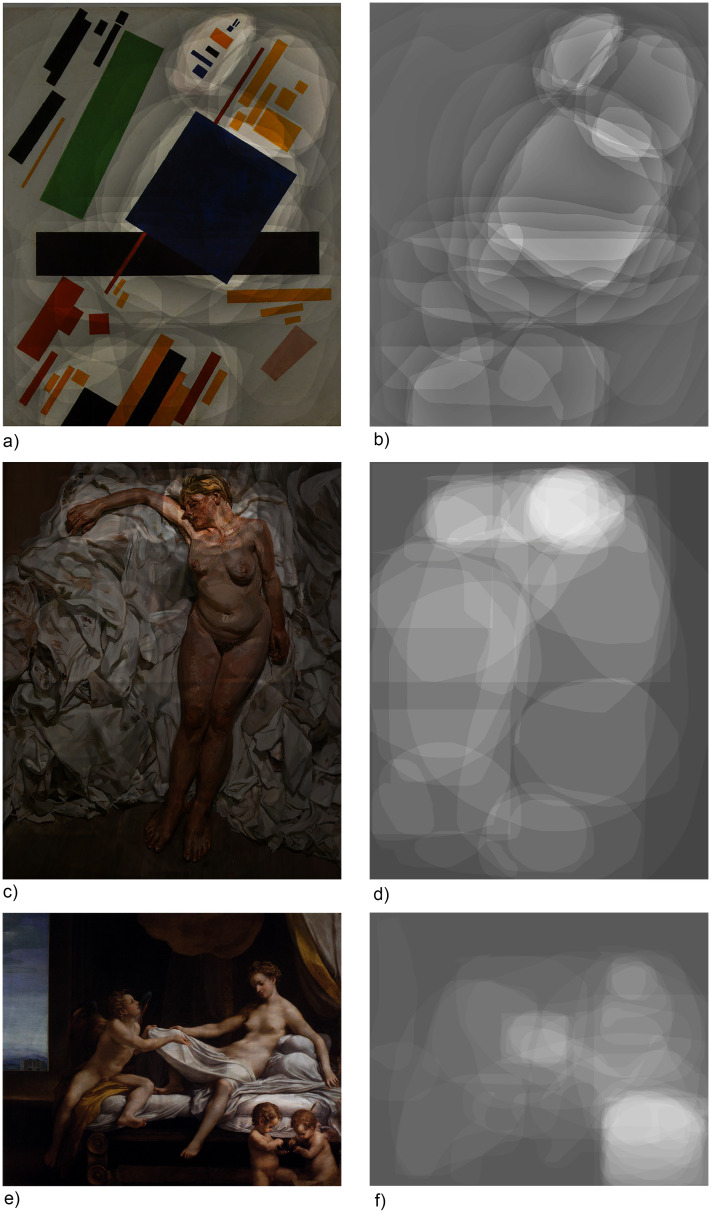
Marked areas by the respondents, indicated by opacity (left: marked areas overlaid on the original painting, right: marked areas only).

Figure 2 presents examples of the average marked areas by all participants for abstract, contemporary, and traditional figural paintings. The illustrations depict the sections of the paintings that were most frequently marked as the most impressive (white sections, referred to as “transparent” areas, indicating regions where the original painting is visible) and those that were never marked (appearing as darkened parts).

The visual analysis of abstract, traditional figural, and modern figural paintings reveals distinct differences in stylistic elements and patterns of attention distribution. Heatmaps of abstract paintings, such as those by Malevich (1913), Rothko (1950), and Richter (1992) (all available in the online folder Paintings), indicate a dispersed focus across the canvas. Areas of higher transparency often align with visually dominant elements, such as the large blue rectangle in Malevich's *Suprematist Composition* or the transitions between forms in Rothko's *White Center*. In traditional figural paintings, including those by Correggio (1531), Velázquez (1634), and Poussin (1648), the heatmaps demonstrate concentrated attention on central narrative elements, particularly human figures and their interactions. For example, in Correggio's *Danaë*, the highest concentration of attention is focused on the central figure of Danaë, particularly on her face and body, as well as on the cherubs at her feet. Similarly, in Velázquez's equestrian portrait, viewers’ attention is drawn to the figure of the Count-Duke, with a specific emphasis on his face and the elaborately detailed armor. In Poussin's *Landscape with Orpheus and Eurydice*, the heatmaps highlight the group of figures in the foreground, particularly Orpheus playing the lyre and Eurydice, whose interaction drives the narrative of the painting. In modern figural paintings, such as those by Freud (1988–1989), Wallinger (1994), and López (1982), the heatmaps display varied patterns of attention distribution, often emphasizing specific areas of visual or emotional prominence. For example, in Freud's *Standing by the Rags*, attention is concentrated on the central nude figure, particularly her face, right hand and upper torso, reflecting the psychological and physical intensity of the depiction. In Wallinger's *Half-Brother*, the heatmaps reveal a focus on the division between the two halves of the horse's body, highlighting the contrast in texture and tonal variation.

The visual inspection highlights how style influences attention distribution. Abstract paintings invite exploration through their forms and colors, traditional figural works direct attention toward narrative and semantic elements, while modern figural works blend these approaches, emphasizing both formal and narrative aspects. This underscores the role of stylistic choices in guiding viewer engagement and interpreting aesthetic elements.

### Descriptive Statistics for Pictorial Measures

The *dimensions of the marked areas* were quantified using three measures: area size, width, and height, expressed as proportions of the entire painting. Each dimension could range from 0 (*No marking*) to 1 (*The entire painting marked*). On average, 17% of a painting was identified as impressive, with an average width of 0.42 and an average height of 0.39 (refer to Table 2).

**Table 2.** Means and SDs (in parentheses) for area, width, height, lab values, and kappas of marked areas.

	All paintings	Abstract	Figural contemporary	Figural traditional
Area (% of total)	0.17 (0.22)	0.20 (0.22)	0.16 (0.22)	0.16 (0.21)
Width (% of total)	0.42 (0.30)	0.50 (0.35)	0.38 (0.27)	0.39 (0.24)
Height (% of total)	0.39 (0.28)	0.41 (0.31)	0.38 (0.28)	0.39 (0.25)
*L*	53.96 (16.96)	60.82 (16.38)	62.13 (13.44)	39.8 (10.06)
*L* range	16 to 99	16 to 88	31 to 99	18 to 65
*L* corrected	0.17 (11.93)	0.13 (12.96)	0.40 (12.81)	0.00 (9.95)
*L* corrected range	−49 to 32	−49 to 23	−28 to 32	−20 to 24
*a*	10.92 (14.83)	19.46 (18.32)	7.02 (12.47)	6.49 (8.44)
*a* range	−5 to 90	−3 to 69	−4 to 90	−5 to 79
*a* corrected	−0.07 (10.23)	−0.15 (11.27)	−0.06 (11.55)	0.00 (7.62)
*a* corrected range	−22 to 78	−22 to 56	−12 to 78	−11 to 73
*b*	19.08 (14.68)	29.87 (17.94)	15.02 (10.47)	12.68 (7.05)
*b* range	−11 to 78	−11 to 77	2 to 78	−10 to 70
*b* corrected	−0.09 (10.62)	−0.11 (14.40)	−0.17 (9.51)	0.00 (6.81)
*b* corrected range	−33 to 59	−33 to 42	−15 to 59	−20 to 55
Fleiss kappa	0.06 (0.05)	0.04 (0.02)	0.06 (0.05)	0.07 (0.06)

**Figure 3.**
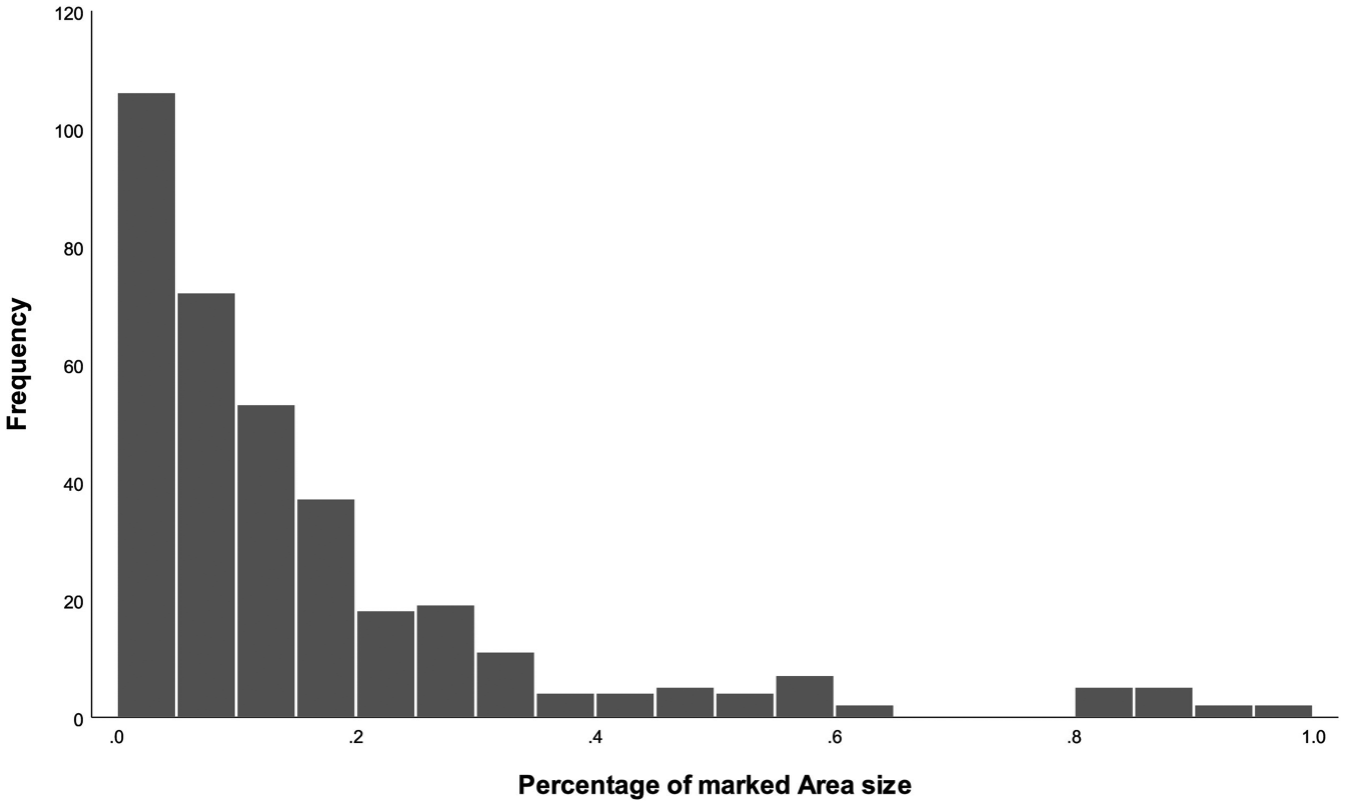
Frequency distribution of the size of marked area.

All marked areas were encompassed by a single shape; there were no instances where someone used two or more fields to indicate the most impressive part of the painting. In 34 cases (9.4%), participants did not mark any part of the painting as impressive, while in 4 cases (1.1%), the entire painting was marked as impressive (see Figure 3).

There were no statistically significant differences in the area size of the marked area among the three painting styles (*F*(2, 78) = 1.96, *p* = .15, *ω*^2^ = .05). Similarly, the differences in the height of the marked area were not significant (*F*(2, 76) = 1.10, *p* = .34, *ω*^2^ = .03). The only significant difference was observed in the width of the marked field (*F*(2, 76) = 8.89, *p* < .001, *ω*^2^ = .19). The marked area of Abstract paintings was significantly wider than the marked areas of the other two styles. In essence, paintings with a more vertical orientation tended to have wider marked areas relative to the total width of the painting.

*Chromatic values* of the marked areas corresponded to the Lab values of the entire painting. For marked areas in Abstract and Figural contemporary paintings the “*L*” values (indicating brightness) were significantly higher compared to Figural traditional paintings (*F*(2, 52) = 62.25, *p* < .001, *ω*^2^ = .71). However, after correcting for the “*L*” value of the marked area by subtracting it from the “*L*” value of the entire image, the differences between the three observed styles disappeared (*F*(2, 52) = 0.15, *p* = .86, *ω*^2^ = .01). A similar trend was observed for the “*a*” (green-red axis) and “*b*” (blue-yellow axis) values, where they matched the “*a*” and “*b*” values of the entire painting, initially suggesting style differences in these values for the marked areas. Nevertheless, after correction, the distinctions in “*a*” and “*b*” values between the three painting styles disappeared (for “*a*” corrected value: *F*(2, 52) = 0.12, *p* = .89, *ω*^2^ = .01 and for “*b*” corrected value: *F*(2, 52) = 0.21, *p* = .81, *ω*^2^ = .01). This pattern indicates that differences in chromatic characteristics within marked areas result from the overall color composition of the paintings rather than inherent differences in the styles themselves.

As expected, there was a significant correlation between the size of the marked area, width, and height (*r_Area-Width_* = .73, *p* < .01 and *r_Area-Height_* = .73, *p* < .01), as well as between height and width *r_Height-Width_* = .52, *p* < .01. For chromatic values, correlations were lower: “*L*” was correlated with “*a*” values *r_L-a_* = .20, *p* < .05 and “*b*” values *r_L-b_* = .16, *p* < .05, but no correlation existed with corrected values. “*a*” and “*b*” values were strongly correlated *r_a-b_* = .62, *p* < .01. The width of the marked area was correlated with the “*L*” value *r_Width-L_* = .22, *p* < .01 and “*a*” value *r_Width-a_* = .20, *p* < .01, a result of the pronounced “squareness” of abstract paintings, which are brighter (“*L*” value) and redder (“*a*” value) (correlation results are shown in Table 3).

**Table 3.** Pearson correlations for pictorial measures.

	Width	Height	*L*	*L* corr	*a*	*a* corr	*b*	*b* corr
Area	.73**	.77**	.08	.07	.10	.04	.04	−.07
Width	1	.52**	.22**	.04	.20**	−.05	.06	−.06
Height	.52**	1	−.05	.06	−.01	.05	.08	−.10
*L*	.22**	−.05	1	.71**	.20**	−.01	.16**	.08
*L* corr	.04	.06	.71**	1	.00	−.01	.09	.12*
*a*	.20**	−.01	.20**	.00	1	.68**	.62**	.37**
*a* corr	−.05	.05	−.01	−.01	.68**	1	.39**	.55**
*b*	.06	.08	.16**	.10	.62**	.39**	1	.72**
*b* corr	−.06	−.10	.08	.12*	.37**	.55**	.72**	1

*Note*. corr = corrected.

*Significant at the 0.05 level (2-tailed), **significant at the 0.01 level (2-tailed).

### Interrater Reliability of Pictorial Measures

Fleiss’ kappa was computed to assess whether there was agreement among participants’ judgments regarding the most impressive aspects of nine paintings. The average kappa value was 0.06, ranging from 0.01 to 0.14. Although the obtained kappa values were significant, their values were very low, suggesting that agreement among respondents is equivalent to chance agreement (Table 2). Overall, the general Fleiss kappa coefficient was very poor, indicating a lack of consensus among participants regarding the most impressive areas of the paintings (Altman, 1990; Landis & Koch, 1977). There were no significant differences in rater agreement among the three styles of paintings (*F*(2, 6)= 0.426, *p* = .671).

### The Relationship Between Pictorial Measures and Aesthetic Preferences

Initially, we investigated whether dimensional (area size, width, and height) and chromatic (*L*, *a*, and *b*) values of Pictorial measures could predict values on three aesthetic scales: Interestingness, Pleasantness, and Comprehensibility. These scales were measured at level 1, nested within each individual, with personal attributes (age, gender, and group membership) measured at level 2. Following the methodological guidance provided by Heck and Thomas (2020), we compared the empty baseline model with random-intercept model, using the Akaike information criterion (AIC). Supplemental materials provide descriptive statistics for aesthetic rating scales, parameter estimates, fit statistics, Mplus syntax, and data files for these models.

The implementation of a random-intercept model (model 1) significantly improved empty baseline model 0, reducing AIC from 3616.4 to 3264.5 (*χ*^2^(27) = 351.9, *p* < .001). The size of the marked area was identified as a significant predictor of Interestingness ratings (standardized *b* = .235, *SE* = .097, *p* = .016) and Comprehensibility ratings (*b* = .279, *SE* = .126, *p* = .027; refer to Table 4). Selection width was found to negatively predict Comprehensibility, a trend attributed to larger width values in abstract paintings. Additionally, the “*a*” value was a significant predictor of Comprehensibility (*b* = −.152, *SE* = .069, *p* = .029), likely due to elevated “*a*” values (redness) in abstract paintings, even when painting style was controlled for. Other Pictorial measures did not demonstrate a significant impact on the aesthetic rating scales.

**Table 4.** Unstandardized and standardized solutions for three models testing the influence of the pictorial measures on aesthetic rating scales.

Model effects	Model 0—empty baseline model				Model 1—random-Intercept with L1 and L2 predictors				Model 2—model 1 with painting style			
	Unstandardized		Standardized		Unstandardized		Standardized		Unstandardized		Standardized	
Model for the mean	Est. (SE)	*p*-value	Est. (SE).	*p*-value	Est. (SE)	*p*-value	Est. (SE).	*p*-value	Est. (SE)	*p*-value	Est. (SE).	*p*-value
Intercept interestingness	4.468 (0.131)	<.001	7.219 (1.430)	<.001	4.655 (0.694)	<.001	8.750 (2.772)	.002	4.727 (0.735)	<.001	8.931 (2.619)	<.001
Slope area size → interestingness					1.578 (0.643)	.014	0.235 (0.097)	.016	1.507 (0.658)	.022	0.224 (0.099)	.024
Slope width → interestingness					−0.804 (0.473)	.089	−0.148 (0.088)	.093	−0.737 (0.463)	.111	−0.135 (0.086)	.115
Slope height → interestingness					0.522 (0.541)	.335	0.093 (0.095)	.332	0.582 (0.532)	.274	0.103 (0.094)	.271
Slope *L* value → interestingness					0.004 (0.008)	.598	0.035 (0.065)	.596	0.004 (0.008)	.614	0.033 (0.066)	.612
Slope *a* value → interestingness					−0.011 (0.010)	.279	−0.074 (0.068)	.276	−0.012 (0.010)	.258	−0.077 (0.068)	.255
Slope *b* value → interestingness					0.009 (0.012)	.483	0.062 (0.088)	.481	0.009 (0.012)	.462	0.065 (0.087)	.460
Abstract painting → interestingness									−0.130 (0.252)	.606	−0.041 (0.079)	.607
Figural contemporary → interestingness									−0.234 (0.223)	.293	−0.073 (0.070)	.296
Fixed effect gender (female reference) → Average interestingness (between)					0.125 (0.237)	.596	0.110 (0.205)	.591	0.122 (0.238)	.608	0.108 (0.209)	.605
Fixed effect age → Average interestingness (between)					−0.007 (0.013)	.589	−0.179 (0.34)	.599	−0.007 (0.013)	.595	−0.177 (0.339)	.601
Fixed effect group (general reference) → Average interestingness (between)					−0.100 (0.415)	.810	−0.086 (0.357)	.810	−0.098 (0.416)	.813	−0.085 (0.359)	.813
Intercept pleasantness	4.330 (0.117)	<.001	8.416 (2.515)	<.001	4.361 (0.637)	<.001	9.461 (6.175)	.125	4.626 (0.657)	<.001	10.627 (5.033)	.035
Slope Area size → pleasantness					1.202 (0.728)	.099	0.178 (0.11)	.106	1.269 (0.638)	.047	0.187 (0.097)	.055
Slope width → pleasantness					−0.175 (0.588)	.766	−0.032 (0.108)	.767	−0.232 (0.54)	.668	−0.042 (0.098)	.669
Slope height → pleasantness					0.034 (0.509)	.946	0.006 (0.09)	.946	0.114 (0.462)	.805	0.02 (0.081)	.804
Slope *L* value → pleasantness					0.008 (0.008)	.291	0.065 (0.061)	.286	0.008 (0.008)	.337	0.06 (0.062)	.335
Slope *a* value → pleasantness					0.01 (0.012)	.394	0.068 (0.079)	.391	0.008 (0.012)	.493	0.053 (0.078)	.491
Slope *b* value → pleasantness					−0.006 (0.014)	.679	−0.040 (0.097)	.679	−0.005 (0.013)	.703	−0.034 (0.089)	.703
Abstract painting → pleasantness									−0.102 (0.215)	.634	−0.032 (0.067)	.635
Figural contemporary → pleasantness									−0.753 (0.200)	<.001	−0.232 (0.064)	<.001
Fixed effect gender (female reference) → average pleasantness (between)					0.244 (0.232)	.293	0.248 (0.191)	.195	0.232 (0.23)	.313	0.249 (0.198)	.208
Fixed effect age → average pleasantness (between)					−0.005 (0.01)	.639	−0.139 (0.32)	.665	−0.005 (0.01)	.623	−0.154 (0.328)	.639
Fixed effect group (general reference) → average pleasantness (between)					−0.153 (0.372)	.680	−0.152 (0.367)	.678	−0.162 (0.374)	.665	−0.171 (0.384)	.657
Intercept comprehensibility	4.372 (0.117)	<.001	9.239 (2.584)	<.001	4.192 (0.491)	<.001	8.020 (2.518)	.001	4.567 (0.433)	<.001	8.479 (1.944)	<.001
Slope area size → Comprehensibility					1.981 (0.900)	.028	0.279 (0.126)	.027	1.17 (0.615)	.057	0.166 (0.088)	.059
Slope width → Comprehensibility					−1.328 (0.521)	.011	−0.23 (0.093)	.013	−0.238 (0.385)	.536	−0.042 (0.068)	.539
Slope height → Comprehensibility					−0.482 (0.499)	.334	−0.081 (0.083)	.332	−0.222 (0.349)	.524	−0.038 (0.059)	.523
Slope *L* value → comprehensibility					0.003 (0.009)	.732	0.022 (0.065)	.733	0.003 (0.007)	.706	0.02 (0.053)	.707
Slope *a* value → comprehensibility					−0.024 (0.011)	.029	−0.152 (0.069)	.027	−0.021 (0.009)	.019	−0.132 (0.056)	.018
Slope *b* value → comprehensibility					0.002 (0.011)	.861	0.013 (0.074)	.861	0.003 (0.008)	.750	0.017 (0.054)	.751
Abstract painting → comprehensibility									−2.176 (0.198)	<.001	−0.649 (0.055)	<.001
Figural contemporary → comprehensibility									−0.374 (0.12)	.002	−0.111 (0.035)	.001
Fixed effect gender (female reference) → average comprehensibility (between)					0.155 (0.242)	.521	0.139 (0.221)	.528	0.15 (0.217)	.489	0.13 (0.191)	.494
Fixed effect age → average comprehensibility (between)					0.015 (0.009)	.105	0.398 (0.25)	.111	0.016 (0.009)	.072	0.404 (0.208)	.052
Fixed effect group (general reference) → average comprehensibility (between)					0.268 (0.355)	.451	0.234 (0.32)	.465	0.24 (0.33)	.468	0.203 (0.283)	.472
*Model for the variance*												
Residual variance for interestingness	2.310 (0.219)	<.001	1 (0)		2.124 (0.211)	<.001	0.941 (0.029)	<.001	2.118 (0.214)	<.001	0.973 (0.07)	<.001
Residual variance for pleasantness	2.246 (0.239)	<.001	1 (0)		2.196 (0.231)	<.001	0.963 (0.025)	<.001	2.112 (0.244)	<.001	0.929 (0.112)	<.001
Residual variance for comprehensibility	2.757 (0.235)	<.001	1 (0)		2.416 (0.210)	<.001	0.958 (0.024)	<.001	1.523 (0.201)	<.001	0.877 (0.114)	<.001
*Initial sad mood variance*												
Random intercept for interestingness	0.383 (0.147)	.009	1 (0)		0.275 (0.141)	.051	0.972 (0.071)	<.001	0.273 (0.126)	.031	0.937 (0.029)	<.001
Random intercept for pleasantness	0.265 (0.154)	.085	1 (0)		0.198 (0.230)	.388	0.932 (0.107)	<.001	0.176 (0.139)	.205	0.913 (0.034)	<.001
Random intercept for comprehensibility	0.224 (0.128)	.080	1 (0)		0.240 (0.160)	.132	0.880 (0.143)	<.001	0.255 (0.1)	.011	0.613 (0.063)	<.001
*Covariance/correlations within*												
Interestingness with comprehensibility	0.783 (0.201)	<.001	0.310 (0.069)	<.001	0.670 (0.183)	<.001	0.296 (0.07)	<.001	0.635 (0.152)	<.001	0.354 (0.062)	<.001
Interestingness with pleasantness	1.364 (0.197)	<.001	0.599 (0.055)	<.001	1.314 (0.18)	<.001	0.608 (0.055)	<.001	1.296 (0.181)	<.001	0.613 (0.053)	<.001
Pleasantness with comprehensibility	0.775 (0.189)	<.001	0.312 (0.063)	<.001	0.734 (0.183)	<.001	0.319 (0.064)	<.001	0.803 (0.162)	<.001	0.448 (0.051)	<.001
*Covariance/correlations between*												
Interestingness with comprehensibility	0.234 (0.09)	<.001	.799 (0.169)	<.001	0.212 (0.093)	.022	0.824 (0.361)	.023	0.221 (0.083)	.008	0.841 (0.146)	<.001
Interestingness with pleasantness	0.259 (0.127)	<.001	.814 (0.138)	<.001	0.197 (0.152)	.195	0.844 (0.258)	.001	0.189 (0.112)	.093	0.862 (0.222)	<.001
Pleasantness with comprehensibility	0.233 (0.092)	<.001	.956 (0.252)	<.001	0.216 (0.092)	.019	0.990 (0.733)	.177	0.203 (0.07)	.004	0.957 (0.215)	<.001

As a control variable in model 2, painting style was included—Abstract and Figural contemporary were significantly less comprehensible compared to Figural traditional (standardized *b_Abstract_* = −.649, *p* < .001 and *b_FigContemp_* = −.111, *p* = .001). Furthermore, Figural contemporary paintings were rated significantly less pleasant compared to Figural traditional (*b* = −.232, *p* < .001). Regarding explanatory power, the *R*^2^ values indicated that Pictorial measures explain 5.9% (*p* = .045) of the variance for Interestingness. However, the explained variances for the other two aesthetic assessments were not significant: 3.7% (*p* = .150) for Pleasantness and 4.2% (*p* = .079) for Comprehensibility at level 1. At level 2, no significant predictors for aesthetic ratings were identified.

## Discussion

The primary objective of this study was to examine the efficacy of a novel technique for assessing aesthetic impressions of visual artworks, termed Pictorial technique. Our analysis of pictorial measures revealed that, on average, approximately 17% of a painting's surface was delineated as “impressive.” The study found minimal differences in the size and chromatic values of marked areas across painting styles. However, the width of marked areas was significantly greater for abstract paintings, likely reflecting their compositional characteristics. In fewer than 10% of the cases, participants did not identify any section of the painting as impressive. Furthermore, respondents consistently utilized a single continuous form to indicate the most striking area within the artwork. The observed low agreement levels among respondents, with only a 6% consensus, suggest a notable lack of consistency in how participants pinpointed these regions. This low concordance underscores the individuality of each observer's pictorial impressions and their varied responses to aesthetic stimuli.

However, a visual inspection of the artworks revealed distinct “islands of transparency,” indicating a relative consistency among observers concerning the most engaging parts of the paintings. Particularly in artworks with representational content, these focal points often correlated with human and animal faces or figures (as seen in works by Freud, Velázquez, Correggio, and Wallinger, detailed in the online Supplemental materials), presumably highlighted due to their significance in conveying emotions, intentions, and social cues. Conversely, in abstract paintings (e.g., works by Rothko, Richter, and Malevich), highlighted sections contributed significantly to the overall composition's dynamism. The marked areas thus exhibit specificity, evidenced by the low observer agreement, yet also a generality, as indicated by a consistent tendency to converge on significant parts of the image. This duality illustrates the nuanced interplay between individual perception and collective tendencies in aesthetic appreciation.

The observed patterns of attention distribution across abstract, traditional figural, and modern figural paintings highlight the complex interplay of composition, style, and content in shaping aesthetic impressions. In abstract paintings, participants’ attention gravitated toward visually dominant elements, such as large geometric shapes, contrasts, and bold colors, as seen in Malevich's *Suprematist Composition* and Rothko's *White Center*. This pattern suggests that, in the absence of explicit narrative or representational content, participants prioritize high-contrast areas, transitions between forms, and chromatic salience. The dispersed nature of attention in abstract works aligns with the idea that viewers rely on formal qualities, such as symmetry, balance, and color interactions, to guide their focus and interpret meaning. For traditional figural paintings, such as those by Correggio, Velázquez, and Poussin, the heatmaps indicate a more concentrated attention distribution. Participants consistently selected central narrative elements, particularly human figures and their interactions. For example, in Correggio's *Danaë*, attention was drawn to the figures of Danaë and cherubs, underscoring their prominence within the narrative. Similarly, in Poussin's *Landscape with Orpheus and Eurydice*, attention focused on the figures engaged in storytelling and action. These patterns underscore the importance of narrative and symbolic content in directing viewers’ attention and shaping their aesthetic experience. Modern figural paintings, such as those by Freud, Wallinger, and López, exhibit a hybrid pattern of attention distribution. While participants focused on central figures, as in Freud's *Standing by the Rags*, there was also notable attention to the surrounding details, such as textures and background elements. This dual focus reflects the interplay between representational content and the compositional emphasis on form and detail in modern works. For instance, in López's *View of Madrid from Torres Blancas*, participants highlighted both the urban landscape and its architectural details, suggesting an appreciation for the compositional complexity of the work.

The patterns observed across the nine paintings emphasize the critical roles of composition, style, and content in shaping aesthetic experiences. In abstract works, attention is guided by the formal properties of the composition, such as contrast and symmetry. In traditional figural paintings, narrative content and the central placement of figures drive attention, reflecting the historical emphasis on storytelling in art. In modern figural paintings, the blending of realism and abstraction creates a dual focus, where viewers engage with both the narrative and the formal qualities of the artwork.

### Possibilities for Integration With Eye-Tracking

These findings highlight the multifaceted nature of aesthetic appreciation, where the interplay of visual, symbolic, and compositional elements directs attention and shapes viewers’ interpretations. Future research could further explore these dynamics by combining PT with physiological techniques, such as eye-tracking, to examine how spontaneous gaze behaviors correlate with reflective aesthetic judgments across different styles and content. One significant advantage of combining eye-tracking with PT is the ability to investigate the interaction between spontaneity and deliberation. Eye-tracking can provide data on viewers’ initial gaze patterns, which are often driven by salience, contrast, and other low-level visual features. These data can be compared with the deliberate selections captured through PT to examine whether participants’ final aesthetic judgments emerge from their initial visual impressions or involve more reflective engagement with the artwork's composition and content. Another advantage of combining these methods is the ability to map attention dynamics over time. By overlaying heatmaps generated from eye-tracking data with those from PT, researchers can observe how attention evolves during the viewing experience. For instance, an artwork may initially attract viewers’ gazes to high-contrast areas or vibrant colors, as captured by eye-tracking, but later elicit more reflective selections that focus on narrative elements or symbolic features during pictorial tasks. This approach can illuminate the temporal dimensions of aesthetic engagement and the progression from automatic perception to thoughtful evaluation. Moreover, eye-tracking data can enhance the understanding of cognitive processes underlying participants’ selections in pictorial measures. Metrics such as scanning patterns, fixation durations, and saccadic movements provide valuable insights into how individuals engage with specific features of an artwork. By comparing these data with participants’ deliberate choices, researchers can identify patterns, such as whether prolonged fixations or repeated revisits to certain areas predict selections of impactful elements. For example, viewers who fixate longer on human figures may also be more likely to select these as aesthetically significant.

### The Relationship Between Pictorial Measures and Aesthetic Preferences

In addition to assessing the validity of the PT in empirical aesthetics, we aimed to test a specific hypothesis regarding the relationship between pictorial measures—particularly the size of the marked areas—and aesthetic preferences. Although the analysis revealed that the size of the marked area significantly predicted responses on the aesthetic scales of Interestingness and Comprehensibility, the explained variance was relatively low, accounting for only 6% and 4%, respectively. This modest effect size raises questions about the practical applicability of this relationship in broader contexts.

These findings suggest that the effect observed in previous studies, which highlighted the relationship between the physical size of aesthetic objects and aesthetic preferences (e.g., Chu et al., 2013; Seidel & Prinz, 2018), is much weaker when applied to the marked areas in the PT. This disparity indicates that the size of the marked area is only marginally associated with aesthetic preferences for the observed paintings. Instead, the results imply that the size of these areas is predominantly determined by the content of the painting, specifically its formal and narrative elements. For example, participants might select larger areas when responding to paintings with complex narratives or intricate compositions, rather than purely based on their aesthetic appeal. Future studies could explore these nuances further by considering additional contextual factors, such as cultural influences or the role of individual differences in aesthetic evaluation, to better understand the determinants of marked areas in PT.

### Limitations of the Current Study

While this study provides valuable insights into the use of the PT as a novel tool in empirical aesthetics, several limitations must be addressed to contextualize the findings and guide future research. (1) *Overemphasis on dominant features.* The PT relies on participants marking specific areas of a painting, which may lack the granularity necessary to capture subtle nuances of visual attention. Unlike methods such as eye-tracking, which provide temporal data and allow for the tracking of dynamic shifts in gaze, the PT only reflects static judgments of what participants consider impactful. This limitation may overlook momentary but significant interactions with less noticeable aspects of the artwork. (2) *Influence of instruction and task framing*. The outcomes of the PT are highly dependent on how the task is framed. Asking participants to mark “the most impactful areas” might elicit responses that are influenced by subjective interpretations of what constitutes “impact.” For instance, some participants may focus on emotional or narrative aspects, while others might prioritize formal qualities such as color or shape. This variability can complicate the interpretation of results and reduce the consistency of findings across studies. (3) *Content-driven selection bias.* The size and location of marked areas are heavily influenced by the content of the artwork, such as its formal composition and narrative elements. For example, in figural paintings, participants are more likely to select human figures or focal points of narrative action, which may overshadow less prominent yet aesthetically significant features. This bias suggests that PT may not fully capture the diversity of elements contributing to aesthetic appreciation, particularly in artworks with complex or non-centralized compositions. (4) *Narrow range of artworks*. The artworks selected for the study represent a limited range of styles and genres, focusing on abstract, traditional figural, and modern figural paintings. While these categories provide a useful framework for initial exploration, they may not capture the diversity of artistic expressions, such as contemporary art, sculpture, or installations. The limited scope may constrain the broader applicability of the findings to other art forms. (5) *Static nature of the PT*. The PT measures participants’ deliberate and reflective selections but does not capture the dynamic, temporal aspects of aesthetic engagement, such as initial impressions or evolving perceptions over time. This static nature limits its ability to provide a comprehensive understanding of how aesthetic experiences unfold during prolonged or repeated exposure to artworks.

Addressing these limitations in future studies could significantly enhance the robustness and applicability of the findings. Expanding the range of artworks, incorporating cross-cultural samples, and integrating physiological measures could provide a more comprehensive understanding of the dynamics of aesthetic experiences.

### Future Research Directions

Integrating the PT into multi-method approaches offers significant potential for advancing the study of aesthetic experiences. Combining PT with methods such as eye-tracking, neural imaging, or verbal protocols can provide a holistic understanding of the interplay between spontaneous visual attention, cognitive processing, and reflective judgments. Future research could explore how PT correlates with physiological techniques to examine temporal and contextual aspects of aesthetic engagement, enhancing the depth and precision of findings. Additionally, PT's adaptability makes it a promising tool for investigating diverse art forms, styles, and cultural contexts, broadening its applicability in empirical aesthetics. Moreover, the adaptability of PT makes it a valuable tool for exploring diverse art forms and cultural contexts. Its flexible application across mediums—ranging from visual art to digital and multimedia works—enables researchers to investigate how cultural and stylistic differences influence aesthetic preferences and interpretations. As a result, PT holds promise as a versatile methodology for examining the complexities of aesthetic appreciation across various domains and populations.

### Conclusion

This study highlights the potential of the PT as an innovative and versatile tool in the field of empirical aesthetics. By enabling participants to identify visually impactful areas within artworks, the PT provides a unique perspective on aesthetic preferences and attention dynamics across diverse styles and genres.

The broader significance of this study lies in its contribution to understanding how viewers interact with visual art. By integrating PT into multi-method research frameworks, future studies can explore how spontaneous visual attention, as captured through methods like eye-tracking, aligns with the deliberate selections recorded by PT. This combined approach offers the potential to deepen insights into the cognitive and emotional processes underlying aesthetic experiences.

The PT represents a valuable addition to the methodological toolkit of empirical aesthetics. Its adaptability for different art forms and cultural contexts, combined with its capacity to reveal participant-driven insights, underscores its utility in advancing the study of art perception and appreciation. This research not only validates the technique's application but also paves the way for further exploration of how it interacts with other physiological and psychological measures to enrich our understanding of aesthetic experiences.

## Supplemental Material

sj-pdf-1-ipe-10.1177_20416695241309780 - Supplemental material for Assessing aesthetic impressions with pictorial measures: A novel approach in empirical aesthetics Supplemental material, sj-pdf-1-ipe-10.1177_20416695241309780 for Assessing aesthetic impressions with pictorial measures: A novel approach in empirical aesthetics by Ivan Z. Stojilović in i-Perception

## Notes

1. We use definition of aesthetic impressions proposed by Augustin and colleagues (2012, p. 188): “the entirety of affective and cognitive results of an aesthetic experience that are object-related and that can at least theoretically be verbally expressed, i.e. be put into aesthetic judgements.”
