# Supplementary material for: Assessing aesthetic impressions with pictorial measures: A novel approach in empirical aesthetics
Source: Iperception. 2025 Feb 14;16(1):20416695241309780. doi: 10.1177/20416695241309780 (PMC11826878; doi:10.1177/20416695241309780)
Supplement: sj-pdf-1-ipe-10.1177_20416695241309780 - Supplemental material for Assessing aesthetic impressions with pictorial measures: A novel approach in empirical aesthetics [file sj-pdf-1-ipe-10.1177_20416695241309780.pdf]

Supplementary materials

Assessing Aesthetic Impressions with Pictorial Measures: A Novel Approach in Experimental Aesthetics

Table 1

List of Utilized Paintings and Their Categories

Author	Title	Year	Style	Motive
Antonio da Correggio	Danaë	1531	Traditional figurative	Human figure
Diego Velázquez	Equestrian Portrait of the Count-Duke of Olivares	1636	Traditional figurative	Animal (horse)
Nicolas Poussin	Landscape with Orpheus and Euridice	1648	Traditional figurative	Landscape
Antonio Lopez	View of Madrid from Torres Blancas	1982	Contemporary figurative	Landscape
Mark Wallinger	Half-brother	1994	Contemporary figurative	Animal (horse)
Lucian Freud	Standing by the Rags	1988-89	Contemporary figurative	Human figure
Kazimir Malevich	Suprematist Composition	1916	Abstract	
Mark Rothko	White centre	1950	Abstract	
Gerhard Richter	Abstract Painting 780-1	1992	Abstract	

Table 3

Means and SDs (in parentheses) for Three Aesthetic Scales and Familiarity

	Interestingness	Pleasantness	Comprehensibility	Familiarity
Abstract	4.40 (1.26)	4.49 (1.00)	2.98 (1.20)	1.68 (0.89)
Figural contemporary	4.39 (0.99)	3.94 (0.89)	4.86 (0.85)	1.63 (0.70)
Figural traditional	4.61 (1.12)	4.57 (1.18)	5.27 (0.73)	2.37 (1.05)

A four one-way repeated measure ANOVAs were conducted to determine whether there was a statistically significant difference in aesthetic ratings (including Familiarity) for three styles of art. The three styles do not differ from each other in their estimated Interestingness F (2, 72) = 0.626, p =.538, partial ω^2 =.02. There are significant differences in the assessment of the Pleasantness of the three styles (F(2, 72) = 5.397, p =.007, partial ω^2 =.13), whereby Figural contemporary style was assessed as significantly less pleasant than the other two styles. Similarly, there are significant differences in the degree of Comprehensibility of the three styles, F(1.54, 55.58) = 98.657, p <.001, partial ω^2 =.73, with the figural styles as the most comprehensible and the Abstract style the least. Finally, there are significant differences in the Familiarity of the three painting styles (F(1.72, 66.89) = 17.297, p <.001, partial ω^2 =.31), with the Figural traditional style being significantly more familiar than the other two styles. These findings highlight distinct impressions and perceptions related to different art styles. While Interestingness is uniformly perceived across styles, Pleasantness, Comprehensibility, and Familiarity vary significantly, offering insights into how different art styles are received and interpreted by viewers.
